# Effect of Nitrite and Nitrate Concentrations on the Performance of AFB-MFC Enriched with High-Strength Synthetic Wastewater

**DOI:** 10.1155/2015/798397

**Published:** 2015-10-01

**Authors:** Jian-sheng Huang, Ping Yang, Chong-ming Li, Yong Guo, Bo Lai, Ye Wang, Li Feng, Yun Zhang

**Affiliations:** ^1^Chongqing Research Institute of Environmental Sciences, Chongqing 401147, China; ^2^School of Architecture and Environment, Sichuan University, Chengdu 610065, China; ^3^School of Chemical Engineering, Sichuan University, Chengdu 610065, China; ^4^Aerospace Wanyuan Industrial Company, Beijing 100076, China

## Abstract

In order to study the effect of nitrite and nitrate on the performance of microbial fuel cell, a system combining an anaerobic fluidized bed (AFB) and a microbial fuel cell (MFC) was employed for high-strength nitrogen-containing synthetic wastewater treatment. Before this study, the AFB-MFC had been used to treat high-strength organic wastewater for about one year in a continuous flow mode. The results showed that when the concentrations of nitrite nitrogen and nitrate nitrogen were increased from 1700 mg/L to 4045 mg/L and 545 mg/L to 1427 mg/L, respectively, the nitrite nitrogen and nitrate nitrogen removal efficiencies were both above 99%; the COD removal efficiency went up from 60.00% to 88.95%; the voltage was about 375 ± 15 mV while the power density was at 70 ± 5 mW/m^2^. However, when the concentrations of nitrite nitrogen and nitrate nitrogen were above 4045 mg/L and 1427 mg/L, respectively, the removal of nitrite nitrogen, nitrate nitrogen, COD, voltage, and power density were decreased to be 86%, 88%, 77%, 180 mV, and 17 mW/m^2^ when nitrite nitrogen and nitrate nitrogen were increased to 4265 mg/L and 1661 mg/L. In addition, the composition of biogas generated in the anode chamber was analyzed by a gas chromatograph. Nitrogen gas, methane, and carbon dioxide were obtained. The results indicated that denitrification happened in anode chamber.

## 1. Introduction

Nitrogen is present in water as inorganic ions and organic compounds. Nitrogenous substances would benefit the growth of the plant. However, natural water will be seriously polluted and become eutrophication if nitrogenous materials are in excess. Eutrophication happens when the concentration of nitrogen, a major factor of eutrophication, exceeds 0.20 mg/L. The aquatic ecosystems will be damaged by the excess nitrogenous substances, which would seriously impact the survival of human and animals. Therefore, it is necessary to reduce nitrogen pollution loads from nonpoint source pollution or point source pollution.

The nitrogenous substances in the wastewater are traditionally removed by nitrification and denitrification [1]. In the last few decades, many other nitrogen removal technologies have been developed such as shortcut nitrification-denitrification [2], simultaneous nitrification and denitrification [3], aerobic denitrification [4], anaerobic ammonium oxidation [5], single reactor high activity ammonia removal over nitrite [6], completely autotrophic ammonium removal over nitrite [7], and oxygen limited autotrophic nitrification-denitrification [8]. Compared to the traditional nitrification-denitrification, the above nitrogen removal technologies receive higher nitrogen removal efficiency. In addition, less reaction time, less oxygen demand, less carbon source, and less operating and infrastructure cost are needed and less sludge is produced [2, 4, 9–11].

MFC technology was developed in the early 21st century. Carbon and nitrogen could be simultaneously removed as well as producing the electricity in MFC. Researchers discovered that the total Kjeldahl nitrogen and nitrate nitrogen could be removed by MFC [12, 13]. There are two different views on nitrogen removal in MFC. One is that the nitrogen is removed in cathode chamber, the nitrate is removed by denitrification [13–16] and electrochemical denitrification [17], and the ammonia and Kjeldahl nitrogen are removed by nitrification and denitrification [12]. In a two-chamber MFC, the ammonium-containing effluent from the carbon-utilizing anode was fed to an external biofilm-based aerobic reactor for nitrification, and then the nitrified liquor was fed to the MFC cathode for denitrification [18]. In dual-cathode MFCs, ammonium nitrogen was removed via nitrification in the aerobic cathode chamber; nitrate was removed in the anoxic cathode chamber [19]. Denitrification occurred simultaneously with nitrification at the cathode chamber [20]. In addition, nitrate was used as an oxidant in the cathode chamber with a daily removal rate of 0.57 mg (NO_3_^−^-N) L^−1^ day^−1^ [21]. The other is that ammonia nitrogen is removed by nitrification in cathode chamber and nitrate is removed by denitrification in anode chamber [22, 23]. In a single chamber air cathode MFC system, over 85% of nitrate was removed [15]. These showed that nitrogen is mainly removed by nitrification, denitrification, and electrochemical denitrification in MFC. But different MFCs have different nitrogen removal mechanisms. The objective of the present research is to study the effect of nitrite and nitrate on the performance of anaerobic fluidized bed microbial fuel cell (AFB-MFC).

## 2. Materials and Methods

### 2.1. Synthetic Wastewater

The wastewater was high-strength nitrogen-containing synthetic wastewater. The chemical components were glucose, sodium bicarbonate, potassium dihydrogen phosphate, ammonium chloride, sodium nitrite, sodium nitrate, and trace metal nutrient. The trace metal nutrient contains Al_2_(SO_4_)_3_·18H_2_O, MgSO_4_·7H_2_O, FeSO_4_·7H_2_O, CaCl_2_·6H_2_O, H_3_BO_3_, (NH_4_)_2_MoO_4_, CoSO_4_·7H_2_O, ZnSO_4_·7H_2_O, CuCl_2_·2H_2_O, NiCl_2_·6H_2_O, and MnSO_4_·H_2_O; their concentrations are 0.10 mg/L, 3.00 mg/L, 3.50 mg/L, 0.30 mg/L, 0.05 mg/L, 0.01 mg/L, 0.30 mg/L, 0.10 mg/L, 0.20 mg/L, 0.01 mg/L, and 0.50 mg/L, respectively [24, 25]. The concentrations of glucose, sodium bicarbonate, ammonium chloride, sodium nitrite, and sodium nitrate were changed during the experiment. The wastewater was replaced on a daily basis and the pH was adjusted to be about 7 by using sodium hydroxide before pumping into the AFB-MFC system.

### 2.2. Reactor System

**Figure 1:**
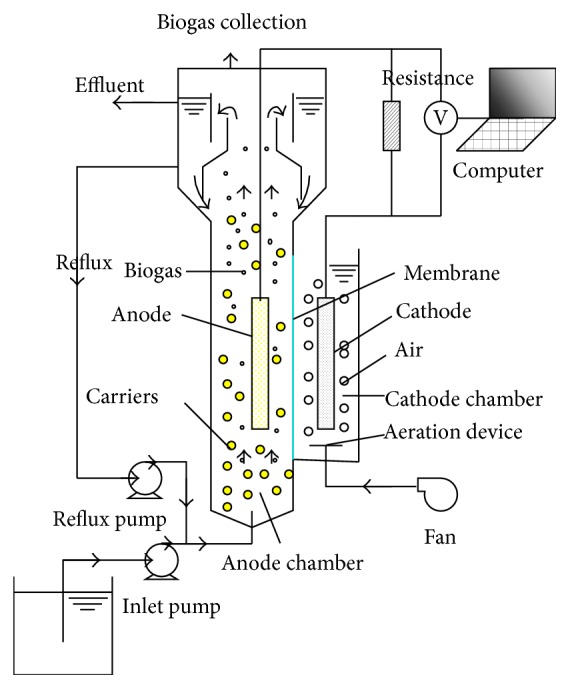
Schematic diagram of AFB-MFC.

The experimental apparatus was a two-chamber MFC system [26]. It consists of an anode chamber (anaerobic fluidized bed with working volume of 7.27 L), a cathode chamber (rectangular vessel with working volume of 1.92 L), and an external resistor. The anode and cathode chambers were separated by a Nafion 117 proton exchange membrane (210 cm^2^, DuPont, USA). The distance between the anode and cathode was 7.0 cm. The anode and cathode were made from carbon fiber paper (Taroy TGP-H-090, with thickness of 0.28 mm). The total surface areas of the anode and cathode were 160 cm^2^ and 210 cm^2^, respectively. A copper wire was used to connect the electrodes to an adjustable resistor (0~99999.9 Ω). Ten percent of the anode chamber volume was added with porous polymer carriers (PPC). The PPC had a dry diameter of 0.32 mm, a wet diameter of 0.56 mm, a grain density of 1320 kg/m^3^, and a wet bulk density of 1010 kg/m^3^. Before this system was used for the present research, the reactor was cultured by anaerobic sludge. It had been successfully started up in a feed-batch mode at a temperature of 35 ± 2°C, pH of 6.8~7.5, aeration rate of cathode chamber of 16~24 L/h, and external resistance of 2000 Ω [24, 25]. Then, it had been used to treat high-strength organic wastewater for about one year in a continuous mode [24, 25]. Bioparticles were formed in anode chamber (Figure 1).

### 2.3. Experimental Procedure

The AFB-MFC reactor was operated in a continuous mode at 35 ± 2°C. The temperature was controlled by a temperature-controlled incubator. The reflux was 10.2 L/h. The aeration rate was 16~24 L/h in the cathode chamber and the dissolved oxygen was about 7.50 mg/L. The external resistance was 120 Ω. The hydraulic retention time (HRT) was twenty-two hours. In the untreated wastewater, the concentrations of glucose and ammonium chloride were 17257 mg/L and 5500 mg/L, respectively. The concentrations of sodium nitrite and sodium nitrate were increased with operation time. The concentrations of COD, ammonium, nitrite nitrogen, nitrate nitrogen, and total nitrogen were examined. Meanwhile, the voltage was recorded by computer. And the composition of biogas produced in anode chamber was analyzed.

### 2.4. Analytical Methods

The COD concentrations of both influent and effluent were measured using a standard method [27]. The pH of the influent, effluent, and the catholyte was measured by a pH meter (Model 618N, JENCO). The ammonia nitrogen, nitrite nitrogen, nitrate nitrogen, and total nitrogen were measured by Nessler's reagent spectrophotometry, N-(1-Nike)-ethylene diamine spectrophotometry, ion selective electrode-flow injection, and Persulfate Oxidation-UV spectrophotometry, respectively [27]. The biological gas from anode chamber was analyzed by a gas chromatograph (GC-2010, SHIMADZU).

## 3. Results and Discussion

### 3.1. Pollutants Removed by the AFB-MFC System

**Figure 2:**
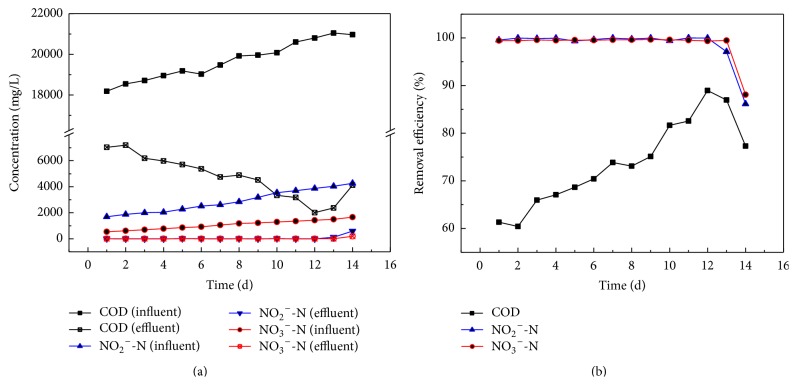
Pollutants removed by MFC system ((a) the concentrations of COD, nitrite, and nitrate in influent and effluent; (b) the removal efficiencies of COD, nitrite, and nitrate).

The pollutants' concentrations of influent and effluent were examined in the experiment. The experimental results were shown in Figure 2. The concentration of nitrite nitrogen was increased from 1700 mg/L to 4045 mg/L while the concentration of nitrate nitrogen was raised from 545 mg/L to 1427 mg/L. The nitrite nitrogen and nitrate nitrogen removal efficiencies were 99.36~99.99% and 99.34~99.67%, respectively. The nitrate nitrogen removal efficiency is higher than that of the literature (85%) [15]. In addition, the COD removal efficiency went up from 60.00% to 88.95%. The results indicated that the level of organic degradation was accelerated when electron accepters, such as nitrite and nitrate, were present in the solution [28]. After 12 days' running, the removal efficiencies of COD, nitrite nitrogen, and nitrate nitrogen were sharply decreased with the increase of the concentrations of nitrite nitrogen and nitrate nitrogen. The results indicated that nitrite nitrogen and nitrate nitrogen were almost completely removed once electron donors were enough. Meanwhile, COD could be efficiently removed. In other words, the presence of electron acceptors such as nitrite and nitrate in the anode increases biodegradation of organic matter, but the concentration of electron acceptors must be appropriate [28].

### 3.2. Electricity Generation

**Figure 3:**
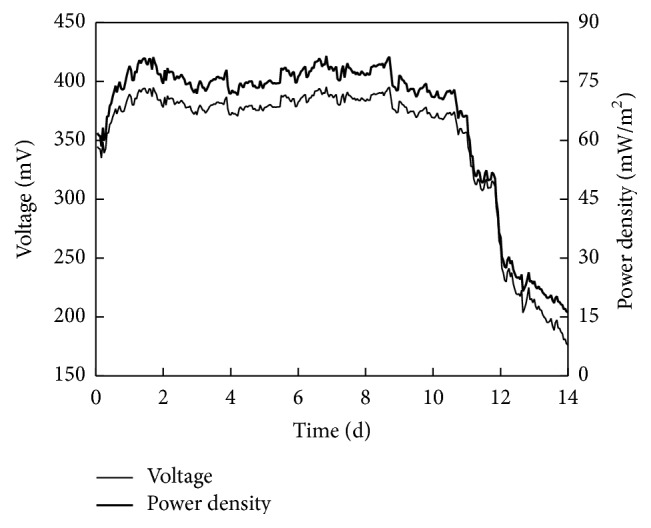
Changes of voltage and power density with operation time.

As shown in Figure 3, the voltage and power density increased with operation time in the first day. It was because of the ionic strength being increased with nitrate and nitrite increasing. Higher voltage and power density were obtained at high ionic strength [29]. From the second day to 12th day, although the COD removal efficiency and the concentrations of nitrate and nitrite increased, the voltage and power density were maintained relatively stable. The voltage, power density, and Coulombic efficiency were about 375 ± 15 mV, 70 ± 5 mW/m^2^, and 1.73*E* − 5%, respectively. This indicated that the amount of electrons used to produce electricity was stable. Then, the voltage and power density decreased within the operation time. In other words, the voltage and power density decreased with the concentrations of nitrate and nitrite increasing. The voltage and power density decreased to 180 mV, 17 mW/m^2^, and 9.14*E* − 6%, respectively. It might be due to the excess of the electron accepters (nitrate and nitrite). The electrons were mainly used by electron accepters. This indicated that proper management of electron accepters was very crucial for obtaining high voltage/power density in a MFC [28].

### 3.3. Analysis of Nitrogen Removal Mechanism

The results mentioned in Section 3.1 demonstrated that the removal is not only limited to the COD, but also to nitrite and nitrate. We proposed a hypothesis that the nitrite nitrogen and nitrate nitrogen in the synthetic wastewater might be removed by denitrification. In order to prove the hypothesis, the concentrations of total nitrogen, ammonia nitrogen, nitrite nitrogen, and nitrate nitrogen in influent and effluent of the AFB-MFC were examined. The results were listed in Table 1. Table 1 showed that ammonia nitrogen concentration of the effluent was appropriately equal to that of the influent. But the concentrations of nitrite nitrogen and nitrate nitrogen of the effluent were extremely lower than those of the influent. The results indicated that the ammonia nitrogen was not removed, but nitrite nitrogen and nitrate nitrogen could be efficiently removed. In the wastewater, the substance that contained nitrogen was ammonia nitrogen, nitrite nitrogen, and nitrate nitrogen. Meanwhile, the concentration of the total nitrogen was almost equal to the sum of the concentration of ammonia nitrogen, nitrite nitrogen, and nitrate nitrogen. The total nitrogen concentration of the effluent was lower than that of the influent. These suggested that nitrogen was removed in AFB-MFC system. In the effluent, the concentration of total nitrogen was approximately equal to the concentration of ammonia nitrogen. Furthermore, the concentrations of nitrite nitrogen and nitrate nitrogen of effluent were extremely lower than that of the influent. These indicated that nitrite nitrogen and nitrate nitrogen were completely removed by AFB-MFC system. As is known to all, nitrite nitrogen and nitrate nitrogen are removed by two roles. One is ammonification and the other is denitrification. In Table 1, it showed that the concentration of ammonia nitrogen of the effluent was approximately equal to that of the influent. These indicated that nitrite nitrogen and nitrate nitrogen were not removed by ammonification but removed by denitrification.

**Figure 4:**
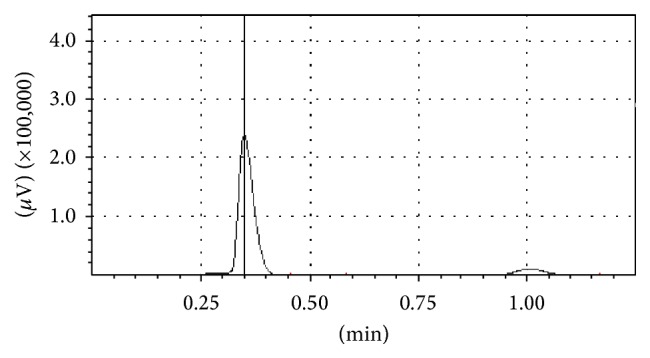
Gas chromatogram (the examination of biogas was done when the influent concentrations of COD, ammonia nitrogen, nitrite nitrogen, and nitrate nitrogen were 20821 mg/L, 1503 mg/L, 3917 mg/L, and 1427 mg/L, resp.; the influent pH was 7.78, and the conductivities of the influent and catholyte were 18.01 mS/cm and 7.17 mS/cm, resp.).

**Figure 5:**
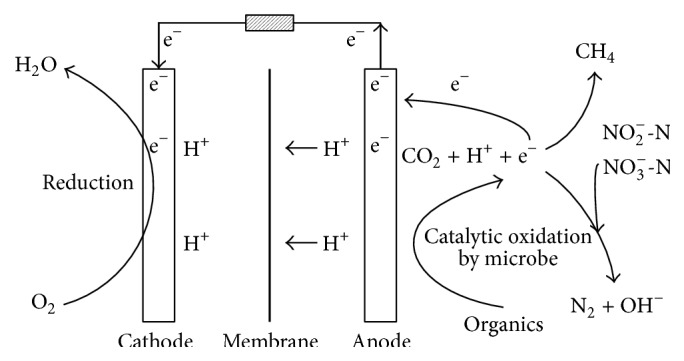
Mechanism of simultaneous electricity generation and carbon and nitrogen removal in AFB-MFC system.

In order to prove that nitrite and nitrate were removed by denitrification, the biogas produced in the anode chamber of AFB-MFC system was analyzed. Firstly, the biogas was collected when the influent concentrations of COD, ammonia nitrogen, nitrite nitrogen, and nitrate nitrogen were 20821 mg/L, 1503 mg/L, 3917 mg/L, and 1427 mg/L, respectively. The corresponding removal rates were 87.50%, 14.41%, 97.59%, and 92.06%, respectively. The influent pH was 7.78, and the conductivities of the influent and catholyte were 18.01 mS/cm and 7.17 mS/cm, respectively. Then, the collected biogas was analyzed by a gas chromatograph, and the nitrogen, methane, and carbon dioxide were qualitatively analyzed. The results (Table 2 and Figure 4) showed that nitrogen, methane, and carbon dioxide were produced in anode chamber during the process of carbon and nitrogen removal and electricity production. Among the three kinds of gases, the proportion of nitrogen, methane, and carbon dioxide was 89.82%, 0.50%, and 9.68%, respectively. It indicated that nitrogen gas was generated. In other words, the nitrite and nitrate were removed by denitrification [15]. Therefore, we provided the mechanism of simultaneous carbon and nitrogen removal and electricity generation of AFB-MFC system (Figure 5). Meanwhile, the reactions that happened in AFB-MFC system were given as follows.

Glucose degradation equation in anode/anode chamber:

(1) $$\begin{array}{c} C_{6}H_{12}O_{6}+6H_{2}O⟶6CO_{2}↑+\;24H^{+}+24e^{-} \end{array}$$

Methanation equation in anode chamber:

(2) $$\begin{array}{c} CO_{2}+8H^{+}+8e^{-}⟶CH_{4}↑+\;2H_{2}O \end{array}$$

Denitrification equation for NO_2_^−^-N in anode chamber:

(3) $$\begin{array}{c} 2N{O_{2}^{-}}^{}+6H^{+}+6e^{-}⟶N_{2}↑+\;2OH^{-}+2H_{2}O \end{array}$$

Denitrification equation for NO_3_^−^-N in anode chamber:

(4) $$\begin{array}{c} 2N{O_{3}^{-}}^{}+10H^{+}+10e^{-}⟶N_{2}↑+\;2OH^{-}+4H_{2}O \end{array}$$

The overall denitrification equation in anode chamber:

(5) $$\begin{array}{c} N{O_{2}^{-}}^{}+N{O_{3}^{-}}^{}+8H^{+}+8e^{-}⟶N_{2}↑+\;2OH^{-}+3H_{2}O \end{array}$$

The reaction equation in cathode:

(6) $$\begin{array}{c} O_{2}+4H^{+}+4e^{-}⟶2H_{2}O \end{array}$$

## 4. Conclusions

The results have demonstrated that nitrite and nitrate nitrogen could be removed by denitrification in anode chamber of AFB-MFC system. The synthetic wastewater with high COD, ammonia nitrogen, nitrite nitrogen, and nitrate nitrogen could be efficiently treated by AFB-MFC system. Meanwhile, proper management of electron accepters was very important for obtaining high voltage/power density in a microbial fuel cell. However, ammonia nitrogen in anode chamber of AFB-MFC system could not be removed. Nitrogen, carbon dioxide, and methane were detected in the biogas which was collected from the anode chamber. These indicated that organic matter was transformed into carbon dioxide and methane, and nitrite and nitrate were transformed into nitrogen gas.

## Figures and Tables

**Table 1:** The substrates' concentration of influent and effluent.

Influent (mg/L)						Effluent (mg/L)					
NH_4_^+^-N	NO_2_^−^-N	NO_3_^−^-N	TN	TN^1^	*R*	NH_4_^+^-N	NO_2_^−^-N	NO_3_^−^-N	TN^#^	TN^1#^	*R* ^#^
1354	1700	545	3669	3599	1.91	1338	7.6	3.1	1399	1349	3.59
1362	2283	860	4595	4505	2.07	1404	14.7	3.8	1460	1422	2.54
1326	2516	927	4865	4769	2.04	1439	8.2	4.3	1471	1452	1.28
1326	2620	1053	5099	4999	1.94	1551	0.9	4.1	1582	1556	1.68
1400	2852	1178	5539	5430	2.00	1540	6.2	4.4	1585	1551	2.18

TN(TN^#^) is the total nitrogen concentration of influent(effluent). TN^1^(TN^1#^) is the sum of ammonia nitrogen, nitrite nitrogen, and nitrate nitrogen concentration of influent(effluent). *R*(*R* ^#^) is the deviation of TN(TN^#^) and TN^1^(TN^1#^). *R*(*R* ^#^) = (TN(TN^#^) − TN^1^(TN^1#^))/TN(TN^#^) *∗* 100.

**Table 2:** Monitoring results of nitrogen, methane, and carbon dioxide composition.

Peak number	Retention time/min	Peak-height	Concentration	Area	Unit	Components
1	0.351	237107.4	89.82	623364.4	%	N_2_
2	0.552	691.9	0.50	2684.3	%	CH_4_
3	1.004	10973.2	9.68	67724.1	%	CO_2_
